# Co‐designing a behavioural intervention for reducing the impact of chemotherapy‐induced peripheral neuropathy symptoms: An evidence‐ and theory‐driven approach

**DOI:** 10.1111/ecc.13671

**Published:** 2022-08-12

**Authors:** Mary Anne Lagmay Tanay, Jo Armes, Catherine Oakley, Liz Bryson, Robin Johnston, Rona Moss‐Morris, Anne Marie Rafferty, Jose Roca, Lesley Sage, Deb Tanner, Lauren Urwin, Toni Wyatt, Glenn Robert

**Affiliations:** ^1^ Florence Nightingale Faculty of Nursing, Midwifery and Palliative Care King's College London London UK; ^2^ School of Health Sciences, Faculty of Health and Medical Sciences University of Surrey Guildford UK; ^3^ Guy's Cancer Guy's and St. Thomas' NHS Foundation Trust London UK; ^4^ Patient Expert; ^5^ Institute of Psychiatry, Psychology and Neuroscience King's College London London UK; ^6^ Oncology and Haematology Rehabilitation Unit Guy's and St. Thomas' NHS Foundation Trust London UK

**Keywords:** cancer, chemotherapy, chemotherapy‐induced peripheral neuropathy, Common Sense Model, experience‐based co‐design, intervention development

## Abstract

**Objective:**

This study aims to co‐design an evidence‐ and theory‐based behavioural intervention to reduce the impact of chemotherapy‐induced peripheral neuropathy (CIPN) symptoms on patients' quality of life.

**Methods:**

Guided by the Medical Research Council Framework for developing and evaluating complex interventions, our intervention development process was guided by (a) findings of systematic reviews, (b) inductive analysis of 39 h of observational fieldwork, 12 patient and 11 clinician interviews, (c) deductive analysis using the Common‐Sense Model to develop a Self‐Regulation Model of CIPN and (d) 17 patients and 18 clinicians co‐designing the intervention.

**Results:**

CIPN perception and coping behaviours were highlighted as processes to target when co‐designing an intervention. The processes targeted in our intervention are CIPN perception and coping behaviours, namely, (a) self‐monitoring of symptoms, (b) communicating and early reporting of symptoms to clinicians, (c) participating in making chemotherapy dose reduction decisions with their clinicians and (d) engaging in self‐management and safety strategies to reduce impact of CIPN symptoms. To address these, a behavioural intervention was deemed suitable.

**Conclusion:**

We developed a self‐regulation model of CIPN and a logic model for documenting the proposed mechanism of action of our co‐designed behavioural intervention for reducing impact of CIPN symptoms.

## 1 INTRODUCTION

Some neurotoxic drugs cause a condition called chemotherapy‐induced peripheral neuropathy (CIPN). There is no drug recommended to prevent CIPN symptoms from developing (Jordan et al., 2020) whilst duloxetine is the only drug that shows efficacy for managing CIPN pain (Loprinzi et al., 2020). Alternatively, chemotherapy doses are sometimes delayed, reduced or discontinued to limit the seriousness of patients' CIPN symptoms (Park et al., 2013).

Previous studies highlight how patients are inadequately prepared to recognise CIPN (Bakitas, 2007; Boehmke & Dickerson, 2005; Tofthagen, 2010a, 2010b). The perceived risk of CIPN as being less important than the risk of cancer (Tanay et al., 2017), perceived need to finish the complete course of chemotherapy (Salgado et al., 2020; Tanay et al., 2017), fear of stopping treatment (Bakitas, 2007; Salgado et al., 2020; Tofthagen, 2010a) and limited understanding of long‐term effects of CIPN (Salgado et al., 2020) influence patients' reporting behaviours and their attitudes towards its management. Furthermore, patients feel inadequately supported to manage their symptoms, realising with hindsight that poorly managed CIPN symptoms negatively impact quality of life (QoL) (Salgado et al., 2020; Tanay et al., 2017; Tanay & Armes, 2019).

Behavioural interventions have been used for managing cancer symptoms such as fatigue (Corbett et al., 2019) and treatment‐related side‐effects (Berry et al., 2014; Given et al., 2008; Hunter et al., 2020) including CIPN (Knoerl et al., 2018, 2019; Tofthagen et al., 2016). They are also shown to modify physical activity behaviours among cancer survivors (Holtzman et al., 2004). The literature shows benefits of behavioural interventions to include influencing or changing cognitions, emotions and behaviours (Daniels, 2015; Newell et al., 2002; Redd et al., 2001).

A systematic review of current evidence found that although behavioural interventions for CIPN exist, the processes and mechanisms of action of how they do or do not work are unclear (Tanay et al., 2021). The Medical Research Council (MRC) Framework for developing and evaluating complex interventions (Craig et al., 2008) highlights the importance of a good theoretical understanding of how an intervention causes change. This is particularly important for complex interventions with several interacting components designed to address problems shaped by the behaviours of both those who deliver and receive the intervention. Involvement of service users at all stages of the development process is also viewed as essential for making it more likely that the intervention can be successfully implemented in practice (Craig et al., 2008). However, current behavioural interventions for CIPN have rarely—if at all—involved stakeholders, for example, patients (recipients of the intervention) and clinicians (who deliver the interventions) in their development process (Tanay et al., 2021).

This study aims to co‐design an evidence‐ and theory‐based behavioural intervention to reduce the impact of CIPN symptoms on patients' quality of life. We (a) describe an evidence and theory‐based approach to co‐designing complex interventions, (b) share our experience of using this approach, (c) present a self‐regulation model of CIPN and (d) propose a behavioural intervention for reducing the impact of CIPN symptoms. We use the guidance for reporting intervention development studies in health research (GUIDED) (Duncan et al., 2020) to structure our findings and the template for intervention description and replication (TIDieR) checklist (Hoffmann et al., 2014) to report and describe the intervention as recommended by GUIDED.

## 2 METHODS

**FIGURE 1:**
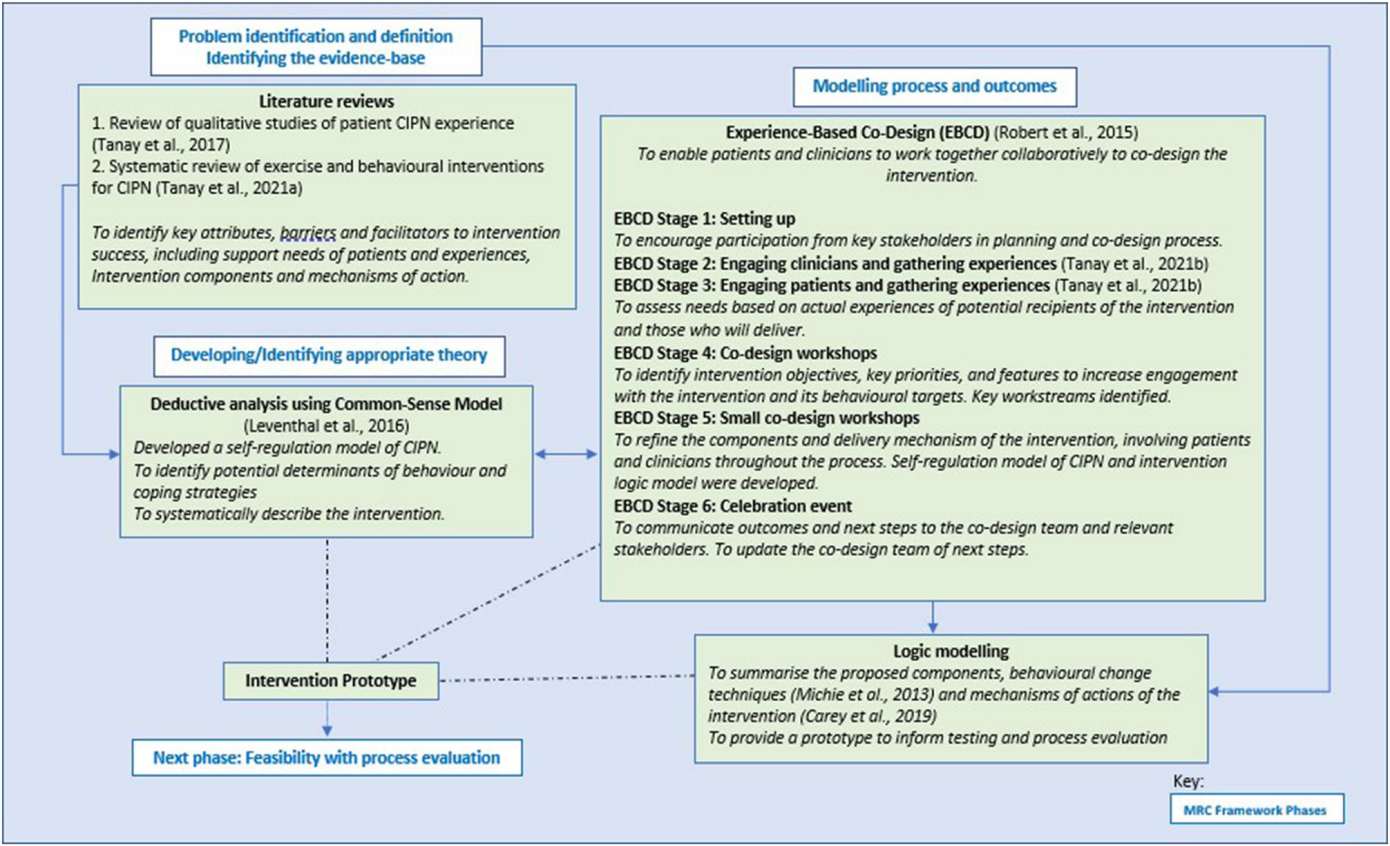
Key elements of the intervention development as aligned with the MRC Framework for developing complex interventions (Craig et al., 2008)

The study was guided by the MRC Framework for developing and evaluating complex interventions (Craig et al., 2008). Building upon a thematic synthesis of CIPN patient experience (Tanay et al., 2017) and systematic review of behavioural interventions in CIPN management (Tanay et al., 2021) previously conducted by the lead author, we employed Experience‐Based Co‐Design (EBCD) (Robert et al., 2015) as a collaborative process to systematically develop the complex intervention with patients and clinicians. This process was informed theoretically by the Common‐Sense Model of Self‐Regulation (Leventhal et al., 2016). Figure 1 provides an overview of the key elements of the intervention co‐design process.

### 2.1 Intervention development using the EBCD approach

#### 2.1.1 Stage 1: Setting up the project

The study was conducted in oncology clinics and a chemotherapy unit at a London cancer centre. Senior clinicians and managers were involved in planning study processes and arranging access to participants. Two patients who had experienced CIPN reviewed the study protocol and ethics applications (referred to as the non‐clinical CIPN expert group or NCEG). East Midlands ‐ Leicester South Research Ethics Committee (REC Reference 19/EM/0192) assessed and approved the study.

Following ethical and site approval, convenience purposive sampling was used to approach potential patient participants. Adults diagnosed with either breast or colorectal cancer, and who were due to receive or who were actively receiving neurotoxic chemotherapy were invited to participate. Clinicians involved in obtaining consent and/or conducting chemotherapy‐related consultations with patients were recruited. All participants signed written informed consent and had the option to attend one or more of the study activities, that is, interview, observation and workshops (see below).

#### 2.1.2 Stage 2 and 3: Engaging patients and clinicians and gathering their experiences

Experience data were collected through observations of chemotherapy patient‐clinician consultations, clinician interviews and filmed/audio recorded patient interviews. Inductive analysis of interview and observation data was carried out. Findings have been published elsewhere (Tanay et al., 2021).

Patient interviews were analysed thematically, edited and compiled by MT into a ‘touchpoint film’. This film explored key themes and issues based on the experiences of people (Tsianakas et al., 2012) who are at risk of or already have CIPN symptoms. Fundamental to the co‐design process, key ‘emotional touchpoints’ in patients' experiences were highlighted in the touchpoint film (Papoulias, 2018).

##### Patient feedback workshop

The workshop was attended by members of the NCEG and study patient participants. Patients who were unable to attend were invited to send their comments by email. The touchpoint film was shown for patients to check for accuracy and resonance with their experiences, as well as to encourage further discussion (member checking) (Birt et al., 2016). An emotional mapping exercise was facilitated by MT where patients shared how they felt as recipients of information and support for CIPN (Donetto et al., 2015) at different points of their treatment journey. Following discussions, participants identified key patient priorities for improvement.

##### Clinician feedback workshop

Thematic analysis of findings from clinician interviews was presented to clinicians, followed by discussion of the broad priorities identified in the interviews and a prioritising exercise. Participants were asked to hypothetically allocate proportions of a set amount of funds according to what they perceived should be prioritised. Further discussions resulted in a set of agreed key clinician priorities.

#### 2.1.3 Stage 4: Joint workshop (co‐design)

Clinician and patient group priorities were presented by a nominated representative from each group. The touchpoint film was then shown, the first instance for clinicians to view the film.

In three mixed patient‐clinician groups, participants discussed the issues presented in the film and group priorities. Each mixed group was given printed materials describing patient and clinician priorities and was asked to discuss and then reprioritise these. They then shared their views with the larger group to agree on a final set of joint priorities.

#### 2.1.4 Stage 5: Small co‐design teams

Specific tasks essential to intervention development were divided among smaller co‐design teams. All participants were grouped into these patient‐clinician teams to work on specific tasks in an iterative design and development process. Graphic designers and a film editor provided creative input.

Stages 2 to 4 took place over 5 months; Stage 5 took 8 months owing to delays caused by COVID‐19 restrictions.

#### 2.1.5 Stage 6: Celebration and progression event

Ten months after the initial joint event, a celebration event was held. This was co‐presented by nominated patient group representatives and clinician group representatives. The purpose of the event was to provide the study update, present the co‐designed intervention, reflect upon the co‐design process and inform participants of the next steps. The event was attended by patient and clinician participants, NCEG and other invited stakeholders who were not directly involved in the co‐design process.

Patients and clinicians were involved as collaborative partners in all stages of the intervention co‐design process, that is, from its inception to final development. All workshops lasted between 1 and 2 h, were audio‐recorded and co‐facilitated by MT, who has extensive facilitation experience in higher education settings. Researcher field notes and workshop transcriptions were analysed, then communicated back to the co‐design teams to inform the focus of both the subsequent workshop and ongoing intervention development.

### 2.2 Theoretical modelling using the Common‐Sense Model

The use of existing theory when developing interventions is proposed within the MRC Framework (Craig et al., 2008). During initial inductive analysis of workshop discussions and comparisons with earlier interview and observation findings, the core constructs from the Common‐Sense Model of Self‐Regulation (CSM) (Leventhal et al., 2016; McAndrew et al., 2008) were recognised as relating to the discussions and outputs. CSM focuses on how individual cognitive and emotional illness representations influence action plans and behaviours for self‐management (Leventhal et al., 2016). Although CSM is largely used to understand perception of illness, for example, cancer, it has been used in research to understand perceptions of key symptoms such as fatigue (Corbett et al., 2016) and pain (Bunzli et al., 2017). CSM research has identified six cognitive illness‐representations dimensions namely: *identity* (beliefs about how the condition is identified or labelled and symptoms associated with the label), *timeline* (beliefs related to how long the illness might last), *consequences* (beliefs about the physical and social effects of the specific health condition on them), *cause* (perceived reason or cause for the development of the illness); *controllability* **(**the individual's beliefs of how much they or their treatment can manage or control the illness and its symptoms) and *illness coherence* (whether the person feels they have a coherent understanding of the illness) (Leventhal et al., 2016; Moss‐Morris et al., 2002).

Following the first joint and three subsequent workshops with smaller co‐design teams, a separate online workshop was attended by patients in which participants validated the key dimensions of CSM with their CIPN experiences. Using CSM as a framework, MT deductively analysed the qualitative data and workshop discussions to understand perceptions of and coping strategies for managing CIPN. Themes were reviewed by GR and JA, followed by confirmation and sense‐checking by patient participants. Our emerging theoretical analysis informed ongoing intervention modelling by the smaller co‐design teams and the structure and process of the co‐design workshops.

### 2.3 Developing a logic model

The MRC Framework recommends a clear description of the intervention and its causal assumptions be provided (Craig et al., 2008). We developed a *logic model* (Kellogg Foundation, 2004) to diagrammatically present a description of the anticipated *intervention components* (the intended activities), *delivery mechanisms* (the structures in place to deliver the intervention), *mechanisms of impact* (our assumptions about how the intervention will produce intended effects) and *intended outcomes*. Behaviour change techniques (BCT) were specified using the Behaviour Change Taxonomy version 1 (Michie et al., 2013) and links between BCTs and mechanisms of action (MoA) were identified using information from a guide developed from a synthesis of BCT‐MoA links as frequently described in the literature (Carey et al., 2019). The task of identifying BCTs and linking these to MoAs was conducted by MT.

Intervention development using the EBCD approach in combination with theoretical modelling using CSM and the development of an intervention logic model was completed within 15 months (longer than initially planned due to the COVID‐19 pandemic).

## 3 RESULTS

### 3.1 Participants

All participants were given the option of attending one or more study activities. In total, there were 42 EBCD participants across all stages. Five patient participants and five clinicians participated in all stages. Four members of the NCEG attended all workshops. Due to COVID‐19 government restrictions, only three initial small co‐design workshops were conducted face‐to‐face; all subsequent workshops (*n* = 7) were conducted virtually. Follow‐up emails were exchanged between members of the co‐design teams after workshops. Table 1 shows demographic characteristics of participants. Table 2 shows involvement according to type and number of participants and by EBCD stage.

**TABLE 1:** Characteristics of EBCD participants

Patient participants								
Study identifier	Gender	Age band	Ethnicity	Cancer diagnosis	Chemotherapy intent	Neurotoxic drug	Chemotherapy treatment stage during data collection	EBCD activity Interviewed (I), observed (O), or attended co‐design workshop/s (CD)
P‐01	Male	70	White	Colon	Adjuvant	Oxaliplatin	Before treatment	I, O, CD
P‐02	Female	40	White	Breast	Adjuvant	Paclitaxel	Midway	O
P‐03	Female	50	Black	Breast	Adjuvant	Paclitaxel	Before treatment	I, O
P‐04	Female	70	White	Breast	Adjuvant	Paclitaxel	Before treatment	I, O
P‐05	Female	50	White	Caecum	Palliative	Oxaliplatin	End of treatment	I, O
P‐07	Female	60	Black	Breast	Adjuvant	Paclitaxel	End of treatment	I
P‐08	Female	60	White	Breast	Adjuvant	Paclitaxel	End of treatment	I, O, CD
P‐09	Female	60	White	Colon	Adjuvant	Oxaliplatin	Midway	I, O
P‐10	Female	70	Black	Colon	Adjuvant	Oxaliplatin	End of treatment	I
P‐11	Female	30	White	Appendix	Adjuvant	Oxaliplatin	End of treatment	I, O, CD
P‐12	Female	30	Mixed white‐Asian	Colon	Adjuvant	Oxaliplatin	End of treatment	I, O, CD
P‐13	Female	60	White	Colon	Palliative	Oxaliplatin	End of treatment	I, CD

Non‐clinical expert CIPN group			
	Gender	Other information	
NCEG 1	Female	Completed neurotoxic treatment	CD
NCEG 2	Female	Completed neurotoxic treatment	CD
NCEG 3	Female	Completed neurotoxic treatment	CD
NCEG 4	Male	Completed neurotoxic treatment	CD

Clinicians			
	Gender	Job role	
C‐01	Female	Cancer nurse specialist	I, O, CD
C‐02	Female	Cancer nurse specialist	O
C‐03	Female	Senior chemotherapy nurse	O
C‐04	Female	Cancer specialist pharmacist	I, CD
C‐05	Male	Cancer specialist pharmacist	I, O, CD
C‐06	Female	Cancer nurse practitioner	I, O
C‐07	Female	Occupational therapist	I
C‐08	Female	Senior chemotherapy nurse	I, O, CD
C‐09	Male	Senior medical oncologist	I, O, CD
C‐10	Female	Senior chemotherapy nurse	IG
C‐11	Female	Senior chemotherapy nurse	IG
C‐12	Female	Junior chemotherapy nurse	IG
C‐13	Female	Senior chemotherapy nurse	IG
C‐14	Male	Junior medical oncologist	I, O
C‐15	Female	Physiotherapist	I, CD
C‐16	Female	Occupational therapist	CD
C‐17	Female	Holistic therapy manager	CD
C‐18	Female	Chemotherapy unit manager	CD

Graphic designers			
	Gender	Role in this project	Specific tasks
GD‐1	Female	Visual illustrator	Production of intervention
GD‐2	Male	Film editor	Production of intervention

Academic researchers	Gender	Role in this research	EBCD tasks
AR‐1	Female	Doctoral researcher	Interviewer, observer, facilitator of co‐design workshops, data analysis (DA), overall study co‐ordination
AR‐2	Male	Co‐design methodology professor	DA, doctoral supervisor
AR‐3	Female	Cancer nurse professor	DA, doctoral supervisor
AR‐4	Female	Behaviour change professor	DA, doctoral supervisor
AR‐5	Female	Workforce and methodologies professor	DA, doctoral supervisor

**TABLE 2:** Types and number of participants and activities of EBCD stages in this study

Stage	Type of participants	Activities
EBCD Stage 1: Setting up	Researcher (MT)	Ethics approval. Engagement with non‐clinical CIPN expert group (NCEG) and clinician collaborators. Meetings with senior clinicians and managers.
EBCD Stages 2 and 3: Interviews	Patients (*n* = 11) Clinicians (*n* = 8) Group interview with clinicians (*n* = 4)	Semi‐structured qualitative interviews. Patient interviews were filmed, analysed and edited by MT into a 20‐min film (touchpoint film).
EBCD Stages 2 and 3: Observations	Patients (*n* = 9) Clinicians (*n* = 8)	Non‐participant observations were undertaken by MT in colorectal and breast cancer clinics and at clinician stations, including the observation of chemotherapy consultations between patients and clinicians.
EBCD Stages 2 and 3: Patient feedback workshop	Patients (*n* = 5) NCEG (*n* = 3)	Group updated about study progress in line with EBCD stages. Touchpoint film was shown to participants. Emotional mapping exercise to identify emotional touchpoints during their chemotherapy journey. Discussions to identify priorities for improving patient experience and attributes of the intervention. Workshop was co‐facilitated by MT and LS.
EBCD Stages 2 and 3: Clinician feedback workshop	Clinicians (*n* = 5)	Group updated about study progress in line with EBCD stages. Summary of qualitative interview findings were presented by CO. Discussions to identify priorities for improving patient experience and attributes of the intervention. Prioritising exercise using a hypothetical fund (£100) to allocate on priority items. Made decisions about key priorities. Workshop was facilitated by MT.
EBCD Stage 4: Joint workshop (co‐design)	Patients (*n* = 5) NCEG (*n* = 3) Clinicians (*n* = 8)	Group updated about study progress in line with EBCD stages. Touchpoint film was shown to participants (first time seen by clinicians). Patient priorities were presented by RJ and another patient participant. Clinician priorities were presented by CO and another colleague. Joint clinician‐patient priorities and workstreams were identified. Workshop was facilitated by MT.
EBCD Stage 5: Small co‐design workshops	Patients (*n* = 5) NCEG (*n* = 4) Clinicians (*n* = 6)	Smaller co‐design groups met for a total of seven workshops. Workshops were followed up with emails in between. One workshop was with patients to discuss and compare alongside their experiences the elements of Common Sense Model. One workshop was with patients, NCEG and a visual illustrator to co‐design visual illustrations of CIPN. Clinicians reviewed and provided opinion on the content and visual illustrations. Ensuing discussions between patients, NCEG and clinicians were through email (due to pandemic restrictions). MT mapped the intervention behaviour change techniques and mechanisms of action Workshops were facilitated by MT.
EBCD Stage 6: Celebration event	Patients (*n* = 3) NCEG (*n* = 4) Clinicians (*n* = 8) Graphic designers (*n* = 1) Other invited clinicians (not part of co‐design team): (*n* = 3) Researchers (*n* = 2)	Presentation of the intervention by DT. LS and RJ, on behalf of patients, shared their experiences of being part of the intervention development process. TW and LU, on behalf of clinicians, shared their experiences of being part of the study. Group discussion at the end of presentations. Closed with announcement of next phase. Event was facilitated by MT.

### 3.2 Identified priorities

Following the separate patient and clinician workshops, priorities for improving patient experience of CIPN were identified by each group. Both groups identified issues in relation to CIPN information provision and the lack of awareness of available resources and opportunities for managing CIPN. Using the SMART criteria (specific, measurable, attainable, relevant and time‐bound) (Blaine Lawlor, 2012), co‐design team members agreed joint priorities. A broad description of an initial intervention prototype was conceived following discussions. Smaller co‐design teams addressed four workstreams: (a) mapping of available information and support for managing CIPN, (b) structuring and collating components of patient information booklet and (c) planning components of film narrative and timing of delivery and (d) working with graphic designers and film editor for booklet layout and film composition.

The joint priority agreed by patients and clinicians guided by the SMART criteria (Blaine Lawlor, 2012) was to develop patient‐informed information materials for patients and also aid clinicians who give information about CIPN to patients. The four identified co‐design workstreams are

- Workstream 1: Mapping of available information and support for managing CIPN. Results will inform the patient information materials.
- Workstream 2: Structuring and collating components of patient information booklet including method/s of delivery.
- Workstream 3: Planning components of film narrative and timing of delivery.
- Workstream 4: Working with graphic designers for booklet layout and film composition.

Additional information of EBCD priorities and workstreams is summarised in Table S1.

### 3.3 A self‐regulation model of CIPN

The Common‐Sense Model (CSM) of Self‐Regulation enhanced our understanding of the complex processes involved in experiences of CIPN and ways to address this condition.

### 3.4 Societal factors

Societal factors that contribute to the interpretative process through which the individual formulates their perception of CIPN were highlighted. How individuals coped with CIPN was influenced by the roles they carry out, for example, at home or in the workplace, and what they perceived to be a general societal lack of awareness of CIPN. Early cognitive and emotional representations of CIPN were influenced by the attitudes of clinicians and the information and descriptions given by them, which coincided with information‐overload about the acute and potentially life‐threatening side‐effects of cancer treatments. Individuals were less in favour of reducing chemotherapy dose for controlling symptoms.

### 3.5 Cognitive representations of CIPN

Attributes of the cognitive representation of CIPN include causal beliefs about symptoms (that influence how the individual labels symptoms and creates identity for CIPN), perceived inevitability of CIPN due to cancer treatment and perceived ability to control the symptoms (which is affected by the individuals' perceived timeline of symptoms). Patient's perceived understanding of CIPN symptoms increased when symptoms were experienced and when symptoms affected activities involving their hands or feet. When symptoms become more noticeable and begin to cause problems, patients also report their symptoms.

### 3.6 Emotional representations of CIPN

The threatening situation, combined with the cognitive beliefs, can trigger emotional distress and low mood, prompting emotion‐focused coping such as minimisation of the problem (not reporting how severe their symptoms were) or avoidance (not reporting their symptoms or avoiding conversations about CIPN). Patients' perception of CIPN was also influenced by fear of death from cancer.

The most common coping strategies individuals used were information‐seeking, positive reframing, seeking social support, active coping and acceptance of the symptoms. However, reappraisal of coping strategies was not observed during their treatment. Rather, patients viewed CIPN as part of an acceptable *new normal* within the context of cancer diagnosis and treatment side‐effects.

**FIGURE 2:**
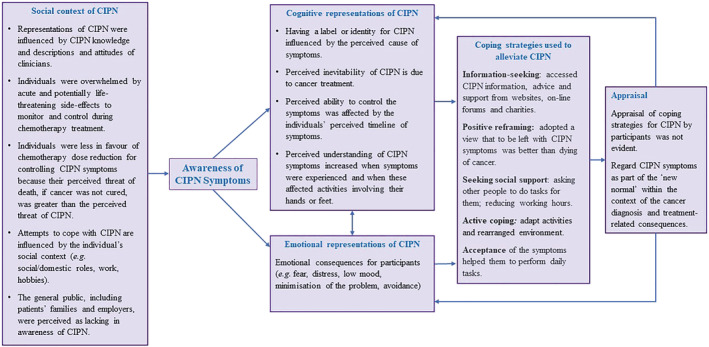
Self‐regulation model of CIPN

Figure 2 illustrates a self‐regulation model to conceptualise CIPN. Exemplars of participant quotes are provided in Table S2.

### 3.7 The REACT‐CIPN intervention

Guided by the CSM, the processes targeted in our intervention are CIPN perception and coping behaviours, namely, (a) self‐monitoring of symptoms, (b) communicating and early reporting of symptoms to clinicians, (c) participating in making chemotherapy dose reduction decisions with their clinicians and (d) engaging in self‐management and safety strategies to reduce impact of CIPN symptoms. We identified potential facilitators of these behaviours and mapped these on to CSM constructs that influence CIPN symptom perception (Table 3). Table 3 also shows sources of evidence upon which we based our assumptions about potential facilitators of behaviours.

**TABLE 3:** Proposed target behaviours for managing CIPN symptoms and facilitators

Target behaviours	Facilitators Patients are more likely to perform behaviour if they: (Evidence: a. thematic synthesis of CIPN patient experience, Tanay et al., 2017; b. systematic review of CIPN behavioural interventions, Tanay et al., 2021; c. clinician and patient interviews and observations, Tanay et al., 2021; d. co‐design activities)	CSM dimension that influence CIPN symptom representation
Self‐monitoring of symptoms	Are encouraged to assess the importance of maintaining function of their hands and feet in relation to their livelihood, domestic roles and hobbies and how CIPN symptoms may affect these. (c, d)	Illness coherence Consequences
	Have understanding and knowledge about CIPN, e.g., acute and long‐term CIPN symptoms and incidence, duration of symptoms, and reason for the development of CIPN. (a, b, c, d)	Identity Timeline Cause
	Are able to envision CIPN symptoms and potential impact on common daily tasks through patient‐informed visual representations. (a, c, d)	Identity Consequences
	Are provided with information that is easily accessible when needed. (b, d)	Identity Consequences
Communicating and early reporting of symptoms to clinicians	Are provided with information about how to recognise early symptoms, when to report and consequences of not reporting. (b, c, d)	Identity Control
	Are encouraged to self‐ monitor symptoms and communicate their symptoms to their clinicians, e.g., note‐taking, what information to mention. (b, c, d)	Timeline Control
Participating in making chemotherapy reduction decisions with their clinicians (if needed)	Are given reinforcement and reminders, i.e., information given at different timepoints of chemotherapy treatment. (c, d)	Identity
	Are encouraged to participate in decision‐making concerning their treatment. (a, c, d)	Control
Engaging in self‐management and safety strategies to reduce impact of CIPN symptoms	Are provided with instructions on how to adapt self‐management and safety strategies. (b, c, d)	Control
	Have increased awareness of available expert support and treatments for managing CIPN symptoms and reducing impact. (b, c, d)	Control
	Are empowered to share information materials with their families, carers, employers and other clinicians such as their GP. (c, d)	Identity Control
	Are motivated to understand and gain control of their own symptoms and engage in self‐management strategies. (c, d)	Illness coherence

To address these behaviours, a behavioural intervention, that is, booklet and film, was deemed suitable. The intervention name agreed by the co‐design team was REACT‐CIPN (Reducing the Impact of Chemotherapy Induced Peripheral Neuropathy). The REACT‐CIPN booklet and film are complementary in targeting behavioural processes.

Behavioural change techniques (BCTs) used in the intervention are (a) information about health consequences, (b) salience of consequences, (c) instruction on how to perform a behaviour and (d) action planning. Proposed BCTs were guided by our findings from the literature reviews (Tanay et al., 2017, 2021), qualitative data analyses and co‐design workshops. For example, boosting perceived CIPN *identity* by providing information about acute and long‐term CIPN symptoms, CIPN incidence and hints on how to recognise early symptoms and when to report these. Visual representations of CIPN symptoms and impact on common daily tasks were also included in the booklet and film. Providing information about adaptive coping safety strategies or self‐management and available expert support can enhance perceived *control (self)* and *control (treatment)*, respectively.

**FIGURE 3:**
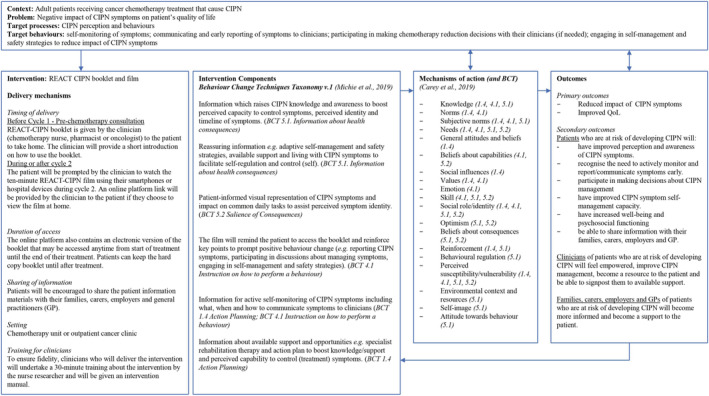
Logic model of REACT‐CIPN intervention components, delivery mechanisms, mechanisms of action and outcomes

Instructions for using the intervention will be given by the clinician before commencing chemotherapy treatment (booklet) and after chemotherapy cycle 2 (film). A logic model showing details of the REACT‐CIPN intervention components, delivery mechanisms, BCTs (Michie et al., 2013) and their mechanisms of action (Carey et al., 2019) and outcomes are illustrated on Figure 3.

## 4 DISCUSSION

The REACT‐CIPN intervention was the direct outcome of a collaborative and theory‐informed co‐design process between patients and clinicians, facilitated by the doctoral researcher. When developing complex interventions, a dynamic, systematic and iterative approach is necessary for illuminating complex processes (Craig et al., 2008; O'Cathain et al., 2019). In our experience, the combination of a theory‐ and evidence‐based co‐design approach was particularly valuable. The MRC Framework (Craig et al., 2008) offers a transparent, methodical and coherent approach to address health needs using research evidence, theory and experiences of patients and clinicians to co‐design a tailored intervention that targets behaviours in a specific patient group. There are several characteristics of our approach that future intervention developers may wish to consider.

The robust evaluation of published research evidence and user experience to underpin the development of an intervention should be considered. From the initial phases of intervention development, our theory‐ and evidence‐based approaches enabled us to (a) gain detailed insight into the context of CIPN experience and patients' needs (Tanay et al., 2017, 2021), (b) identify mechanisms of action of current behavioural interventions for reducing CIPN symptoms and potential barriers to intervention success (Tanay et al., 2021) and (c) incorporate existing knowledge into our intervention. Previous research shows that illness perceptions can act as mediators between illness activity and coping styles (Diefenbach & Leventhal, 1996). We have drawn on the CSM to interpret empirical determinants of CIPN perceptions into theoretical constructs. To our knowledge, no research has applied the self‐regulation model to understand the subjective experiences of patients with CIPN.

In our study, several social responses to CIPN symptoms were shown to have influenced cognitive and emotional representations. For example, the lack of societal CIPN awareness implies that for many patients, their initial perception of CIPN formed when they were told (for the first time) by their clinicians about all potential side‐effects of their chemotherapy treatment. CIPN information provided by clinicians is key to developing CIPN perceptions; thus, timing must be considered. Strategies such as reinforcement of CIPN information at cycle 2 onwards, availability of information in formats preferred by patients and easily accessible CIPN information were suggested.

Aside from recognising the symptoms and impact (*identity*) of CIPN, the patient's perception of CIPN will influence interpretation of the *cause* of the symptoms. This is particularly challenging because the threat of cancer is clearly more important than eliminating the cause of CIPN, that is, the chemotherapy that could save their lives. The *consequences* of continuing or stopping chemotherapy will be internalised and made part of the person's CIPN representation and resulting behaviours. Further, perceived *control* of CIPN symptoms will influence reporting or self‐managing behaviours which can affect symptom severity.

In addition, a person's ability to change the overall *timeline* or duration of symptoms, with respect to reporting or making chemotherapy dose reduction decisions and self‐management, will be an important component of the person's CIPN representation. The ongoing interaction between internal (i.e., impact of CIPN on quality of life) and external factors (i.e., clinician and public opinion) changes the illness representation over time and further guides the individual's actions in response to the threat (CIPN) (Diefenbach & Leventhal, 1996; Leventhal et al., 2016). Emotions (e.g., fear, anger, distress, low mood and avoidance) in CIPN also contribute towards formation of illness representation and develop together with the cognitive component (Diefenbach & Leventhal, 1996, Leventhal et al., 2016). These emotions can motivate the individual to create an action plan or coping strategy. On the other hand, they can be overwhelming and lead to limited, or lack of action or coping. This theoretical understanding of the CIPN experience helped us to recognise target behavioural processes and select appropriate behaviour change techniques aiming to promote target behaviours.

Complementing theory‐ and evidence‐based approaches, the EBCD approach ensured patient needs and perspectives were understood and clinicians' perspectives were accommodated to maximise engagement and future implementation. Although EBCD has been used predominantly for quality improvement (Green et al., 2020), this was not the first instance of this approach being used in developing complex interventions in cancer care (Tsianakas et al., 2015). Co‐design approaches encourage patients to impart their perspective and voice and allow them to meaningfully reflect upon their shared experiences with their clinicians. Instead of being passive sources of information, patients become active partners in prompting change (Robert et al., 2015). Realignment of power dynamics between service users (patients) and service providers (clinicians) and sustained involvement of each as partners in a change process are key to successful co‐design in healthcare (Green et al., 2020). Whilst we faced several challenges—including restrictions due to the COVID‐19 pandemic—we were able to maintain momentum and retained sufficient patients and clinicians in all six stages of EBCD. The flexible nature and level of involvement in various stages of the EBCD process helped to maintain involvement and reduced attrition. We also emphasise the role of the researcher as facilitator and mediator in maintaining momentum. The importance of facilitation in co‐design stages has been highlighted in the past, particularly when joint priorities are set and co‐design activities are planned (Green et al., 2020).

We acknowledge limitations of this study. First, combining purposive sampling and the option to engage in chosen study activities may have resulted in a sample that was not representative of the wider population of patients with CIPN. Despite efforts to include interview participants from ethnic minority backgrounds in the co‐design activities, no participants from this group of interviewees consented to participate. This is a common observation in healthcare studies including participatory health research (Roura et al., 2021). To address this, the researchers ensured that data from their qualitative interviews (Tanay et al., 2021) were considered by the co‐design teams. Patient participants in the co‐design teams who reviewed intervention information ensured these were presented in plain English and that no medical jargon was used. Visual images showing hands and feet were inclusive and portrayed diverse races and genders. Secondly, patients frustrated with CIPN‐related issues might have been more likely to participate in the study and all activities. Conversely, clinicians who were particularly interested in the topic and so were motivated to improve CIPN practice and services were more likely to join. The collaborative and user‐centred nature of the EBCD approach enabled participants to reflect on their shared experiences, to identify issues and to explore potential solutions together. Finally, the first author of this paper analysed the data and was also the facilitator in the workshops. Previous experiences, personal beliefs and objectives and cultural background of the researcher could bias analysis and reporting (Green & Thorogood, 2018). However, efforts were made to minimise this bias by continuous sense checking with the participants. Additionally, four patient and four clinician members of the co‐design teams were involved in co‐authoring this article.

## 5 CONCLUSION

The study showed that the EBCD approach can usefully complement theory‐based and evidence‐based intervention development. It provided ongoing opportunities for patient and clinician participants to work collaboratively in co‐designing a behavioural intervention aimed at reducing the impact of CIPN symptoms. Guided by the newly developed self‐regulation model of CIPN, the two processes targeted in our intervention are CIPN perception and coping behaviours. To address these, a behavioural intervention containing four behaviour change techniques was deemed suitable. We developed an intervention logic model vital for documenting the proposed mechanisms of action of the co‐designed intervention. This model will be refined and tested in a subsequent process evaluation as part of a feasibility trial of the intervention.

## CONFLICT OF INTEREST

The authors declare that there is no conflict of interest.

## Supporting information


**Data S1.** Supporting Information

## Data Availability

The datasets during and/or analysed during the current study are available from the corresponding author on reasonable request. The authors have full control of all primary data which is available upon request.
