# Supplementary material for: Co‐designing a behavioural intervention for reducing the impact of chemotherapy‐induced peripheral neuropathy symptoms: An evidence‐ and theory‐driven approach
Source: Eur J Cancer Care (Engl). 2022 Aug 12;31(6):e13671. doi: 10.1111/ecc.13671 (PMC9786800; doi:10.1111/ecc.13671)
Supplement: Supplementary file 1 — Data S1. Supporting Information [file ECC-31-e13671-s001.pdf]

Co-designing a behavioural intervention for reducing the impact of chemotherapy-induced peripheral neuropathy symptoms: an evidence- and theory-driven approach

Tanay MAL, Armes J, Oakley C, Bryson E, Johnston R, Moss-Morris R, Rafferty AM, Roca J, Sage L, Tanner D, Urwin L, Wyatt T, Robert G

Corresponding author: Tanay MAL mary.tanay@kcl.ac.uk

European Journal of Cancer Care

Supporting information

Figure 1 Supporting Information. Screenshots of the REACT-CIPN intervention

A. REACT-CIPN Booklet

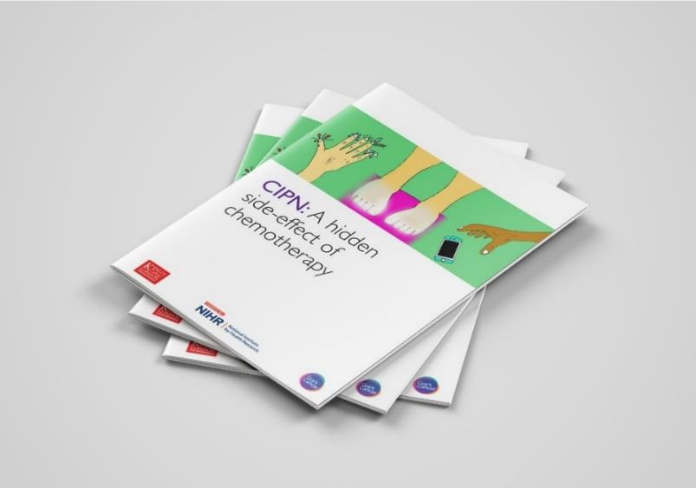

B. REACT-CIPN Film

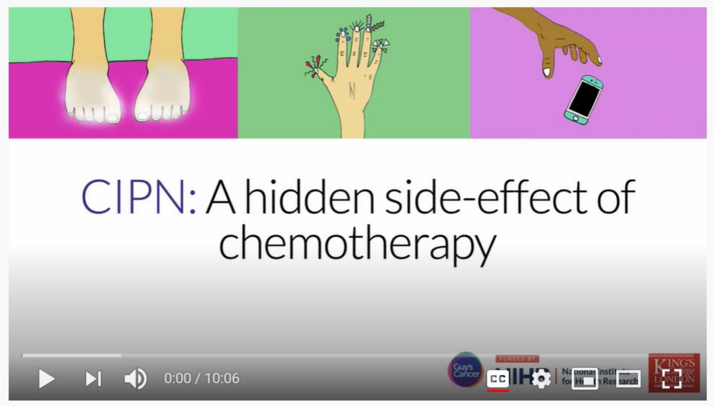

Table 1 Supporting Information. Summary of EBCD priorities and workstreams

		**Possible action**	**Proposed CIPN intervention attributes by co-design participants**
**Patient-identified priorities**	Patients do not retain CIPN information.	Improve ways of providing helpful CIPN patient information	**Content:**<br>− Explains CIPN in words that describe the symptoms well – use examples of patient experiences; broaden the language not just tingling, spell out the various forms of CIPN and how symptoms affect an individual<br>− Gives clear information of the differences between acute and long-term CIPN symptoms and impact<br>− Provides clarity about temporality versus permanence of symptoms<br>− Highlights how symptoms may affect individual hobbies, daily activities and career/livelihood<br>− Suggests practical tips for managing symptoms<br>− Provides information about CIPN support available and who to contact<br>− Gives balanced information about risk of developing CIPN and completing treatment<br>− Encourages patients to participate in making decisions about reducing or stopping treatment<br>− Provides guidance for early reporting of CIPN symptoms and how to communicate these to clinicians<br>− Provides reminders about CIPN symptoms and what to watch out for throughout the chemotherapy treatment<br>− Includes voices of persons who have experienced CIPN<br><br>**Design and layout of written information/leaflet:**<br>− Highlights key information succinctly and visually<br>− Uses visual representation of incidence to help inform likelihood of having CIPN<br>− Provides easily accessible format, depending on patient's preference, such as hard copy booklet or online access<br>− Allows space for recording questions patients may have for their clinicians about CIPN
	Patients' perceived lack of awareness about CIPN among carers, friends and employers.	Provide CIPN information for carers, families and employers.	− Provides resources that can be shared with carers, friends and employers to raise awareness without exaggerating

	Patients’ perceived lack of support for managing CIPN after treatment.	Consider support available for managing CIPN after treatment.	− Provides group classes or support groups for CIPN
	Patients’ perceived lack of CIPN knowledge and understanding, particularly symptom-management, among clinicians.	Provide CIPN education for clinicians, including GPs.	− Ensures patient involvement when developing patient information material
**Clinician identified priorities**	No standard CIPN information provided to patients.	Improve ways of providing CIPN patient information.	− Develop patient information resources that are consistent and in multiple formats<br>− Provide information about CIPN which should include risk, nature of CIPN symptoms, prevention and management<br>− Identify the best time to provide CIPN information<br>− Encourage patient-led monitoring and reporting<br>− Provide information which should cover all stages from pre-chemotherapy until follow-up and what to report in every stage
	Need for education and training for clinicians about CIPN.	Provide learning opportunities for clinicians about CIPN.	− Develop evidence-based CIPN protocol/guidance<br>− Develop a video or film about CIPN and how to prevent or manage symptoms
	Lack of awareness of support for managing CIPN provided by other departments.	Expanding the service with members of the MDT involved.	− Improve communication between clinicians<br>− Develop strategy for early referral to CIPN rehabilitation services<br>− Provide clinicians reminders about available CIPN support and services (for regular and new staff)<br>− Expand service capabilities
**Agreed joint priorities guided by SMART criteria (Blaine Lawlor, 2012)**		To develop patient-informed information materials for patients and also aid clinicians who give information about CIPN to patients.	
**Co-design workstreams**<br><br>− Workstream 1: Mapping of available information and support for managing CIPN. Results will inform the patient information materials.<br>− Workstream 2: Structuring and collating components of patient information booklet including method/s of delivery.<br>− Workstream 3: Planning components of film narrative and timing of delivery.<br>− Workstream 4: Working with graphic designers for booklet layout and film composition.			

Supplementary Table 2. Exemplars of patient quotes (all names are pseudonyms)

Themes	Patient quotes and conversations during workshops
Social context of CIPN	"Neuropathy, no-one really understood my hands were a bit numb, you know, take notes at meetings, use the computer a lot and pretend everything's fine, just get on and you're alive. So it probably comes back to you later when you're through the cancer and then you think, God I'm stuck with this, I used to be able to play the guitar...If anything neuropathy's downplayed a little bit because with everything else that's going on, you know, people know about sickness, fatigue, hair loss, everything else, neuropathy's probably underplayed a little bit. I think actually healthcare professionals, they know about neuropathy, but they don't actually know what it's like unless they happen to have been through it themselves… How do you support the decision making when you can't explain really what neuropathy's like?"" (John, 60 y/o)<br><br>"So there was only the one person I noticed who talked about her career, was that XX that she was a set designer and would need her hands. And that was one of the things that certainly struck me now living with it all these years, is that if I was reliant on my hands, whether I was a bricklayer or a carpenter or a concert pianist I'd be scuppered. And if I was a builder and relied on confidence through my legs to go up scaffolding, I'd be equally scuppered." (Fran, 70 y/o)<br><br>"How much is work educating healthcare professionals including the GPs?" (Fran, 70 y/o)
Cognitive Representations of CIPN	"So you'll live if you have the full whack but you're going to suffer with neuropathy. So you've got to have the choice really haven't you, you cannot say, 'I don't want it'." (Fred, 60 y/o)<br><br>"If they'd said to you before your treatment, right we recommend chemo because it'll clean out the cancer but you will be left with numbness in your hands and feet, you don't really know what numbness, you don't know what it means.so … But if you've got the information right about neuropathy and if you got more informed information about dose reduction, you actually could then make a value judgement…I don't know about the other drugs but with Oxaliplatin I think they confuse the tingling while you're having the drug and the cycles, to the long-term neuropathy which is a completely different thing. The cold with the tingling that's during your chemo. They mash the two up when they're talking to you about symptoms… I think it would have been maybe useful to talk to someone who'd been through it beforehand to actually explain a bit better, rather than a brochure or a staff person who hadn't actually had it. Or maybe even given leaflets about it, maybe add some verbatims in from patients or if it was online little video clip, trying to explain what it's like." (John, 60 y/o)<br><br>"I noticed after my last round of chemo because I don't tend to feel right usually, when I try to write, it's like a child…I couldn't really control it. But because I use a computer at work it hasn't really affected me, it was only when I was trying to write Christmas cards that I noticed, I asked someone else to do it for me. But thankfully it wasn't an issue in my job." (Lisa, 30 y/o)<br><br>"CIPN seems like a fair price to pay to be alive…I think it's interesting that, and it probably happens to a lot of people that if you're just told it's tingling you think, oh that's absolutely fine. But as it gets worse maybe not so fine, and people like XX who've had two lots of chemo that there's a kind of build up." (Molly, 60 y/o)<br><br>*(Conversation about CIPN severity)*<br>John: But again you don't even know what that scale is.<br>Molly: I don't know what it is, not a clue.<br>Joan: Mine was put down at some point, 2 to 3, level 2 to 3 or grade 2 to 3 but I don't know who came up with that.

	Molly: It's so subjective that it's not measurable in the way that if your eyesight's failing somebody can put a finger on it,<br>Lisa: Because I don't really like trust my judgement of my own body anymore because I was so ill but I was like, how would I have not known I was so ill so I'm kind of like, is my neuropathy bad or good.<br>John: How do you know what scale you're …<br>Lisa: No you don't.<br>John: Yours might be far worse than mine and mine might be far worse than yours but we don't know that. (laughs)<br>Molly: No you could do it on tasks I suppose, you could do it on can you do X, Y and Z. But sometimes you can and sometimes you can't.
Emotional representations of CIPN	"I've got to be honest I held back, you know, with the symptoms, you go in there and say, oh I feel great, wonderful, the fittest man on the planet (laughs) wonderful but it's not true, you're frightened that they're going to cut the dose and you're not going to be here. " (Fred, 60 y/o)<br><br>"I think the lady who talked about, felt more depressed now than she did at the cancer, at the diagnosis… as you begin to appreciate what it's going to demand of you living with it year in, year out. And if you are told you're going to die and then you end up not dying, which has happened to Molly and me, once you begin to realise you haven't started to die yet and you think well actually, I'm going to have to live with this for a long time so I have to get to grips with this and make it work for me. And you know that's an exceptionally wonderful feeling to be able to experience but I can appreciate that for a lot of patients that are on their own and not got support then that could bring a sense of being low hearted, depressed." (Fran, 70 y/o)<br><br>"But it's the invisible things that actually more the mental health impact because nobody's sort of offering you a seat on the train because you're wearing a bandana because clearly you've lost your hair. They don't know that there's this thing going on with you...I think it is hard to put forward because with everything that's going on a bit of numbness is like the least of your worries, you know, but it is what you're left with. You worry about death …" (John, 60 y/o)<br><br>"Yeah because I think risk of death, I'm thinking, o my gosh – the risk of death!" (Lisa, 30 y/o)<br><br>"Well, we could have known some of those suggestions up front." (Molly, 60 y/o)<br><br>"So that's then a lack of information that we're given to take charge of it in the first place." (Fran, 70 y/o)
Coping strategies used to alleviate CIPN	"It's never right and never will come back and I think it's a standard thing to tell you. Your new normal expression is a thing that we all have. (Molly, 60 y/o)<br><br>"Kept it where it was. But a friend of mine who plays in a band, plays bass guitar, and she had it in her fingers, she actually refused her final chemo because she said she could not live without being able to play." (Joan, 70 y/o)<br><br>"Understanding neuropathy is the key." (John, 60 y/o)

Key: y/o = years old

Table 3 Supporting Information. Completed guidance for reporting intervention development studies in health research (GUIDED) checklist

**Item description**	**Page in manuscript where item is located**	**Other***
1.Report the context for which the intervention was developed.	1-2	
2. Report the purpose of the intervention development process.	3-6	
3. Report the target population for the intervention development process.	3	
4. Report how any published intervention development approach contributed to the development process	3-6	
5. Report how evidence from different sources informed the intervention development process.	3-7	
6. Report how/if published theory informed the intervention development process.	5-6	
7. Report any use of components from an existing intervention in the current intervention development process.	NA	
8. Report any guiding principles, people or factors that were prioritised when making decisions during the intervention development process.	3-7	
9. Report how stakeholders contributed to the intervention development process.	3-11	
10. Report how the intervention changed in content and format from the start of the intervention development process	8-16	
11. Report any changes to interventions required or likely to be required for subgroups.	NA	
12. Report important uncertainties at the end of the intervention development process.	20	
13. Follow TIDieR guidance when describing the developed intervention.	Supplement	
14. Report the intervention development process in an open access format.		
Item 5 evidence published elsewhere:<br>Tanay, M. a. L., Armes, J., Moss-Morris, R., Rafferty, A. M. & Robert, G. 2021a. A systematic review of behavioural and exercise interventions for the prevention and management of chemotherapy-induced peripheral neuropathy symptoms. *Journal of Cancer Survivorship*.<br>Tanay, M. a. L., Armes, J. & Ream, E. 2017. The experience of chemotherapy-induced peripheral neuropathy in adult cancer patients: a qualitative thematic synthesis. *Eur J Cancer Care (Engl)*, 26.<br>Tanay, M. a. L., Robert, G., Rafferty, A. M., Moss-Morris, R. & Armes, J. 2021b. Clinician and patient experiences when providing and receiving information and support for managing chemotherapy-induced peripheral neuropathy: A qualitative multiple methods study. *Eur J Cancer Care (Engl)***,** e13517.		

Adapted from: Duncan, E., et al., *Guidance for reporting intervention development studies in health research (GUIDED): an evidence-based consensus study*. BMJ Open, 2020. **10** (4): p. e033516.

Table 4 Supporting Information. Completed template for intervention description and replication (TIDieR) checklist

**Item No.**	**Description**	**Item**	**Reported in this paper**
1	Brief name	Provide the name or a phrase that describes the intervention	Yes
2	Why	Describe any rationale, theory, or goal of the elements essential to the intervention	Yes
3	What	Materials: Describe any physical or informational materials used in the intervention, including those provided to participants or used in intervention delivery or in training of intervention providers. Provide information on where the materials can be accessed (such as online appendix, URL)	Yes
4		Procedures: Describe each of the procedures, activities, and/or processes used in the intervention, including any enabling or support activities	Yes
5	Who provided	For each category of intervention provider (such as psychologist, nursing assistant), describe their expertise, background, and any specific training given	Yes
6	How	Describe the modes of delivery (such as face to face or by some other mechanism, such as internet or telephone) of the intervention and whether it was provided individually or in a group	Yes
7	Where	Describe the type(s) of location(s) where the intervention occurred, including any necessary infrastructure or relevant features	Yes
8	When and How Much	Describe the number of times the intervention was delivered and over what period of time including the number of sessions, their schedule, and their duration, intensity, or dose	Yes
9	Tailoring	If the intervention was planned to be personalised, titrated or adapted, then describe what, why, when, and how	NA
10	Modifications	If the intervention was modified during the course of the study, describe the changes (what, why, when, and how)	NA
11	How well	Planned: If intervention adherence or fidelity was assessed, describe how and by whom, and if any strategies were used to maintain or improve fidelity, describe them	NA
12		Actual: If intervention adherence or fidelity was assessed, describe the extent to which the intervention was delivered as planned	NA

Adapted from: Hoffmann, T.C., et al., *Better reporting of interventions: template for intervention description and replication (TIDieR) checklist and guide.* BMJ : British Medical Journal, 2014. **348**: p. g1687.
